# The Suppression of Spurious Modes in TC-SAW Resonators by the Application of Bent Metal Strips

**DOI:** 10.3390/s25226926

**Published:** 2025-11-13

**Authors:** Menghui Li, Mengke Qi, Yuanhang Chen, Yimin Cheng, Liang Cao, Hong Zhou, Xiaojing Mu

**Affiliations:** 1 Key Laboratory of Optoelectronic Technology and Systems of Ministry of Education, International Research and Development Center of Micro-Nano Systems and New Materials Technology, Chongqing University, Chongqing 400044, China; limenghui@stu.cqu.edu.cn (M.L.); caoliang_26@cqu.edu.cn (L.C.); 2 Ministry of Education Key Laboratory of Micro and Nano Systems for Aerospace, School of Mechanical Engineering, Northwestern Polytechnical University, Xi’an 710072, China; zhouhong@nwpu.edu.cn

**Keywords:** surface acoustic wave, resonators, transverse modes, temperature compensation, velocity, gap mode

## Abstract

This article investigates the use of bent metal strips on the top of a SiO_2_ layer for the suppression of spurious modes in temperature-compensated surface acoustic wave (TC-SAW) resonators employing a SiO_2_/Cu/128°YX-LiNbO_3_ structure. The proposed metal strip method includes two parts: a primary metal strip located at the edge of the interdigital transducer (IDT) aperture region and a secondary metal strip in the gap region. The impact of the geometric parameters of bent metal strips was calculated by the 3D finite element method (FEM), and theoretical simulation results show that this method can effectively suppress the transverse modes and mitigate the gap modes originating from the gap region in conventional TC-SAW resonators. Furthermore, experimental validation further confirms that the proposed method can effectively suppress nearly all spurious modes without degrading the performance of the quality factor.

## 1. Introduction

With the rapid development of mobile communication technology, especially the advent of carrier aggregation (CA) and fifth-generation (5G) communication, the demand for high-performance acoustic devices is rising rapidly [1,2,3]. In order to meet the requirements of high-quality communication and large transmission data volumes, acoustic devices with wide bandwidth, low insert loss, low-temperature drift, high out-of-band rejection and improved *Q* factors have been widely recognized in the field of RF filtering [4,5,6,7]. In comparison with bulk acoustic wave (BAW) devices, standard piezoelectric SAW devices using piezoelectric single crystal substrates are cheaper and compact but exhibit higher frequency drift characteristics over temperature [8], resulting in a deterioration in insertion loss and out-of-band rejection in filters [7]. A solution called TC-SAW was developed by combining a piezoelectric substrate and a lower thermal expansion layer to achieve the aim of low-temperature drift [9,10,11,12].

In addition to the challenge of frequency drift with temperature, another inevitable challenge for SAW devices is transverse modes, which are caused by the layered structure and waveguide nature of the busbars [9,13]. The transverse modes often exist in the passband of resonators and result in excessive ripples and performance deterioration in the passband of the synthesized filter. Several methods have been developed to remove the transverse modes in SAW resonators, such as the selective removal of SiO_2_ [14,15], a bent structure [13], the piston technique [16,17], IDT apodization [18], a tilted electrode structure [19,20], and other technical approaches [9,21,22,23,24]. Moreover, another spurious mode called the “gap mode” generated between the aperture region and busbar region will cause a strong response near the anti-resonance and further result in strong nonlinear signals [25,26]. The method of the partial thinning of SiO_2_ in the gap region was proposed to suppress the gap mode [27], but the precise control of the etching profile poses significant challenges for mass production.

This article proposes a method called bent metal strips located at the top of the SiO_2_ layer, wherein the top metal strips located at the edge of the IDT aperture can suppress the generation of the transverse mode by piston mode operation; the top metal strips located at the gap region can reduce the SAW velocity in this region and reduce the scattering effect, thereby mitigating the gap mode. The article is constructed as follows. First, the response of conventional TC-SAW resonators was calculated by 3D periodic FEM, and the results show that transverse modes caused by energy propagation in the lateral direction are serious. The impact of the geometric parameters of bent metal strips on transverse modes was analyzed by 3D FEM, including the height of metal strips and the length of metal strips at the edge of the IDT aperture and gap region. Finally, the experimental results further confirm that the proposed method can effectively suppress nearly all spurious modes without degradation in the performance.

## 2. Modeling and Simulation

### 2.1. Transverse Modes in Conventional TC-SAW Resonators

**Figure 1:**
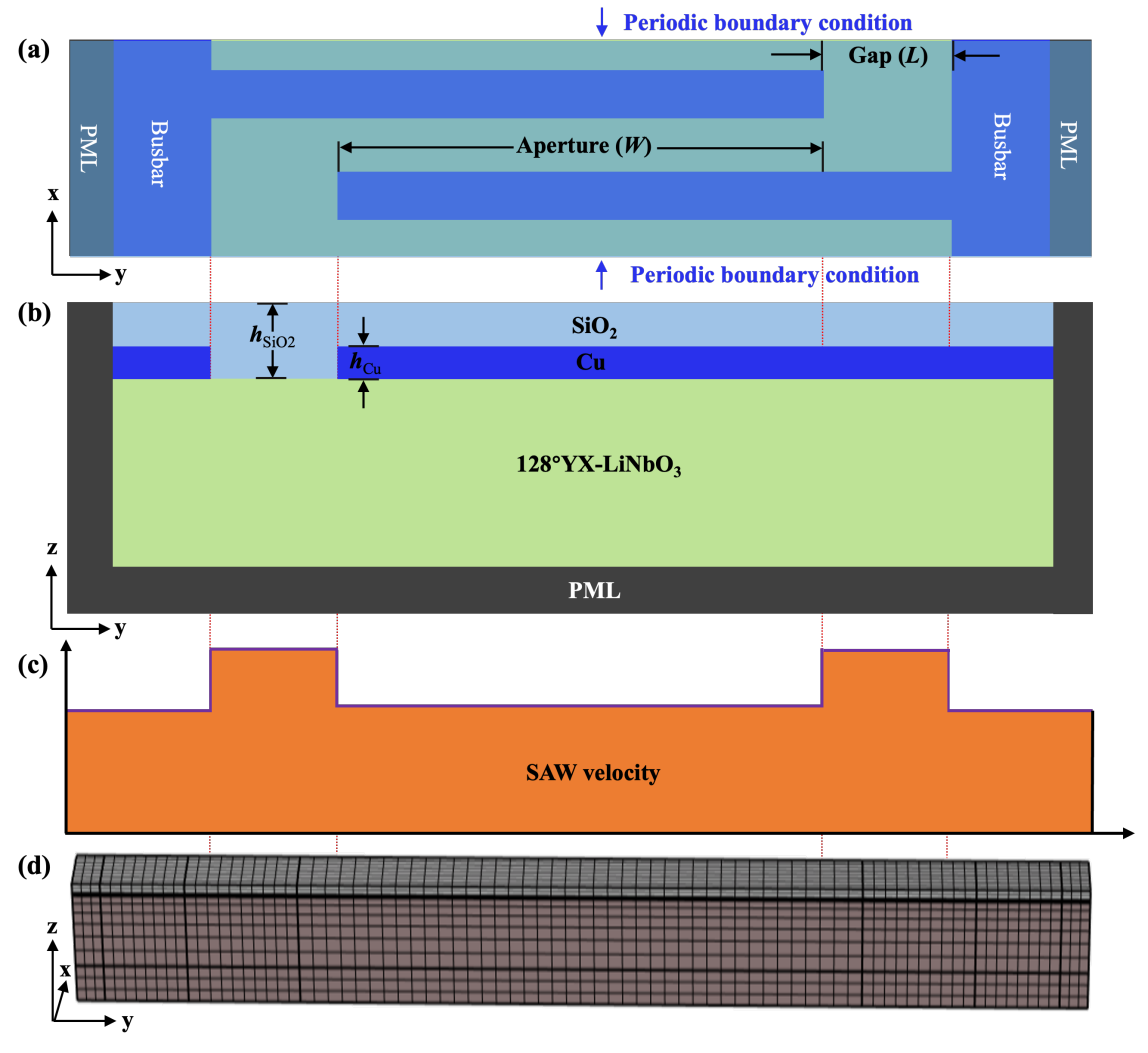
The unit cell of conventional SiO_2_/Cu/128°YX-LiNbO_3_ TC-SAW resonators used for the calculation. (**a**) Top view. (**b**) Cross-sectional view. (**c**) The calculated SAW velocity at different regions along the aperture direction. (**d**) The mesh of the 3D periodic model in COMSOL software.

Top and cross-sectional views of the conventional SiO_2_/Cu/128°YX-LiNbO_3_ TC-SAW resonator unit cell used for calculation are shown in Figure 1a,b, where the *x* and *y* directions represent the longitudinal and transverse directions, respectively. Since the main mode of the TC-SAW resonator is Rayleigh mode, the long gap region will be adopted to provide better energy confinement for the Rayleigh mode to achieve a higher *Q* factor [28,29]. Figure 1c shows the calculated SAW velocity profile corresponding to distinct regions along the aperture direction by FEM eigen-frequency solver [30,31], indicating that the velocity at the gap region is higher than that in other regions and the velocity barrier is constructed between the gap and aperture region.

The response in conventional 3D periodic TC-SAW resonators was calculated using the frequency domain solver of the COMSOL Multiphysics 6.2 software. In the following calculations, the perfect matching layer (PML) is applied at the bottom and side edges of the unit cell structure to absorb the leaky acoustic waves, while periodic boundary conditions are imposed to side boundaries in the *x* direction [24]. The Euler angle of 128°YX−LiNbO_3_ in COMSOL Multiphysics 6.2 software is set as (0°, −38°, 0°), and small material loss (0.001) is applied to the piezoelectric substrate. The electrical conditions of a pair of IDT electrodes in the periodic unit cell are set to ground and 1 V, respectively. In addition, the design parameters utilized in the following calculations are listed in Table 1 and the material constants of LiNbO_3_ and SiO_2_ are taken from [32,33]. As shown in Figure 1d, the constructed 3D model is meshed with the shape of cubic elements. Since the displacement of the surface acoustic wave is at its maximum near the surface, the domain near the surface is discretized to a higher density than the bottom. In addition, the electrode structure in the model is covered by the top SiO_2_ layer.

**Figure 2:**
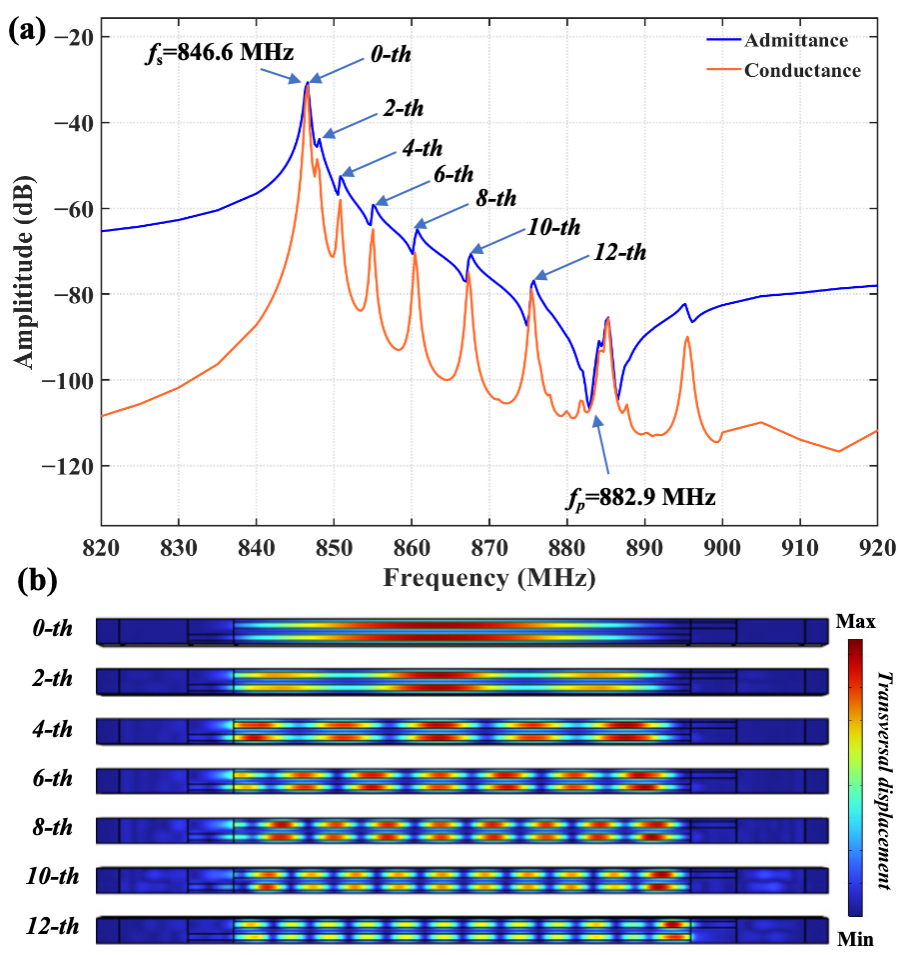
(**a**) The calculated admittance and conductance of the conventional SiO_2_/128°YX-LiNbO_3_ TC-SAW resonator. (**b**) The calculated field displacement distribution of the conventional TC-SAW resonator.

Figure 2a shows the calculated admittance and conductance (Y&G) of the conventional 128°Y-X LiNbO_3_ TC-SAW resonator in Figure 1, and it can be noticed that the transverse modes between *f_s_* and *f_p_* are obvious. Figure 2b shows the calculated transversal displacement distribution for transverse modes marked by arrows in Figure 2a, which unveils a transverse resonance mode caused by the lateral propagation of acoustic energy in the aperture region. It is worth noting that the displacement distribution in the transverse direction is not symmetric, which may be due to the anisotropy of the LiNbO_3_ material, a phenomenon that has previously been reported in SAW devices based on LiTaO_3_ [34].

### 2.2. The Method of Bent Metal Strips for the Suppression of Spurious Modes

**Figure 3:**
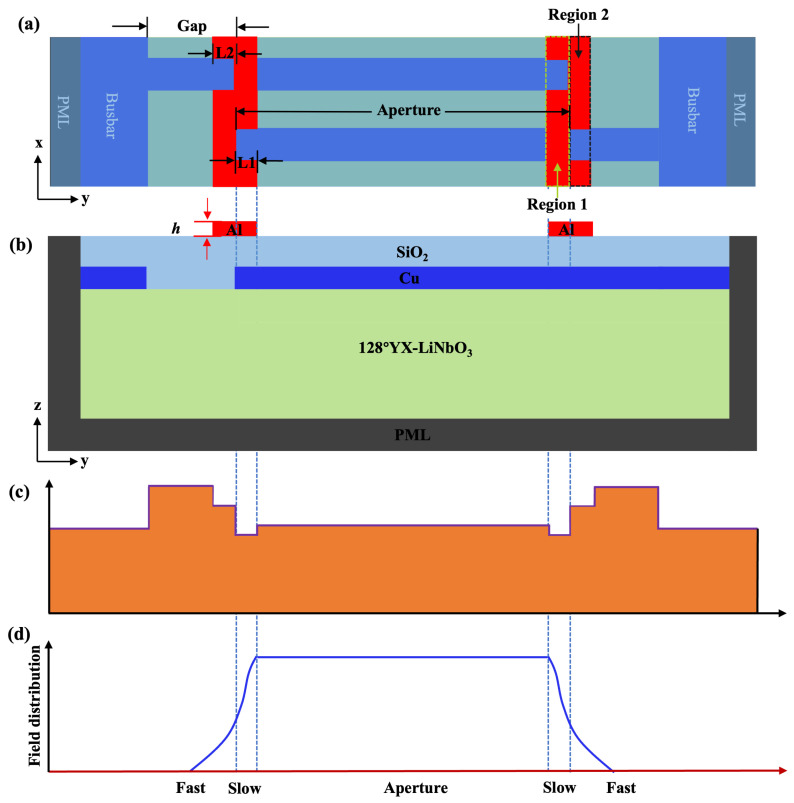
The unit cell of the SiO_2_/128°YX-LiNbO_3_ TC-SAW resonator with bent metal strips on the top of the SiO_2_ layer. (**a**) Top view. (**b**) Cross-sectional view and (**c**) calculated SAW velocity profile at different regions along the aperture direction in the proposed TC-SAW resonator. (**d**) The schematic diagrams of the acoustic field distribution along the aperture direction of the TC-SAW resonator with the proposed method.

In order to suppress the transverse modes generated in conventional TC-SAW resonators, the bent metal strip method shown in Figure 3 was proposed to achieve better suppression for these unwanted spurious modes. Figure 3a,b show the top and cross-sectional view of the schematic diagrams of the proposed structure, respectively. It is noteworthy that the bent metal strips on the top of the SiO_2_ layer include two parts: the primary metal strip located at the edge of IDT aperture region called region 1 and the secondary metal strip in the gap region called region 2. Figure 3c shows the SAW velocity profile corresponding to distinct regions along the aperture direction calculated by the FEM eigen-frequency solver [30], indicating that the bent metal strips on SiO_2_ will reduce the corresponding velocity in region 1 and region 2, respectively, compared with the conventional TC-SAW structure shown in Figure 1.

In accordance with the previously reported methods called piston mode [25,28], the introduction of the region with slower velocity at the edge of IDT electrodes (region 1) will change the distribution of the acoustic field from the excitation source throughout the active aperture region. The optimized acoustic field distribution shown in Figure 3d makes the overall vibration mode of the transducer closer to that of an ideal “piston”, where the entire surface moves with the same phase and amplitude. This “piston” mode of vibration preferentially and efficiently excites the main modes while suppressing the higher-order transverse modes that require non-uniform vibration to be generated [17]. It should be noted that the the piston mode will be effectively excited when the velocity and size of the slow region in the SAW resonator satisfy the Equations (1)–(2) [17].

(1) $$\frac{L_{1}f}{v_{IDT}}≅\sqrt{1+\gamma }\frac{\arctan\left(\sqrt{-\frac{\Delta_{F}}{\Delta_{S}}}\right)}{2\pi \sqrt{-2\Delta_{S}}}$$

where $L_{1}$ is the length of the slow region, $v_{IDT}$ is the acoustic velocity in the IDT aperture region, *f* is the frequency, γ is the anisotropy factor, and $\Delta_{F}$ and $\Delta_{S}$ are the normalized velocity differences between the fast velocity and slow velocity regions and the IDT region, respectively.

(2) $$\left\{\begin{array}{c} \nu_{S}=v_{IDT}\left(1+\Delta_{S}\right)\\ \nu_{F}=v_{IDT}\left(1+\Delta_{F}\right) \end{array}\right.$$

Meanwhile, the resulting larger velocity barrier by piston mode between region 1 and the outer region is beneficial for the confinement of the energy within the aperture region. However, it should be noted that this large velocity barrier will lead to the scattering effect of acoustic waves at the interface [35], which in turn generates an undesired gap mode [27]. Therefore, the metal strips located at region 2 are necessary as they will reduce the SAW velocity there, which in turn reduces the velocity barrier between regions 1 and 2. This will minimize the acoustic wave scattering effect and mitigate the generation of the gap mode.

### 2.3. Optimization of Geometric Parameters for Bent Metal Strips

**Figure 4:**
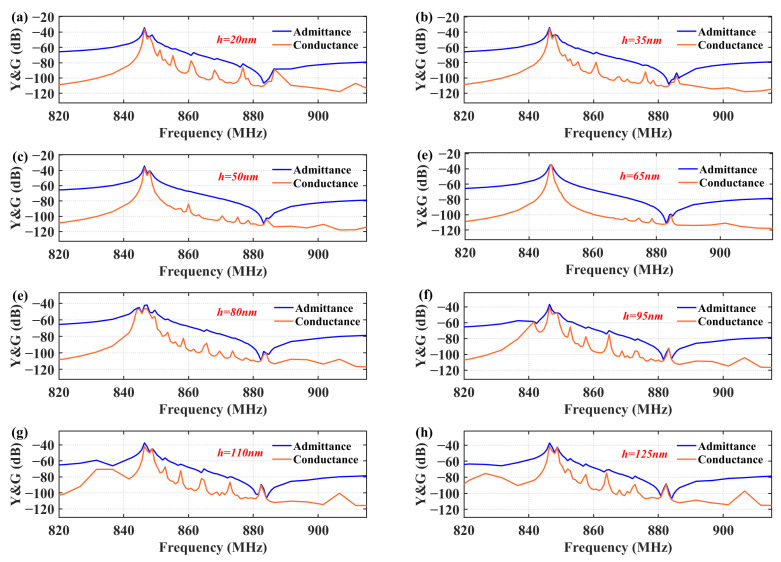
Calculated admittance and conductance curves of TC-SAW resonator with bent metal strip on the top of SiO_2_ under different metal thicknesses. (**a**) 20 nm; (**b**) 35 nm; (**c**) 50 nm; (**d**) 65 nm; (**e**) 80 nm; (**f**) 95 nm; (**g**) 110 nm; (**h**) 125 nm.

The impact of geometric parameters of bent metal strips for spurious modes was calculated by 3D FEM, including the thicknesses of metal strips *h* and the length of metal strips at the edge of the IDT aperture *L*_1_ and gap region *L*_2_. Figure 4 shows the impact of the thickness of the metal strips on transverse modes in the TC-SAW resonator when the length of *L*_1_ and *L*_2_ are fixed at 1.25 μm. When the value of *h* increases, the strength of the transverse modes gradually decreases. When the thickness of the metal strip reaches 65 nm, the transverse modes are well suppressed and only a few spurious modes remain near the anti-resonance, which proves that the piston modes are efficiently excited at the thickness of 65 nm. However, if the thickness continues to increase beyond 65 nm, the performance of the resonator deteriorates again. Transverse modes reappear, and unwanted split emerges below resonance due to the increased metal thickness reducing the SAW velocity in that region, which in turn prevents the efficient excitation of piston modes. Based on the above analysis, the thickness of the bent metal strips is selected to be 65 nm.

**Figure 5:**
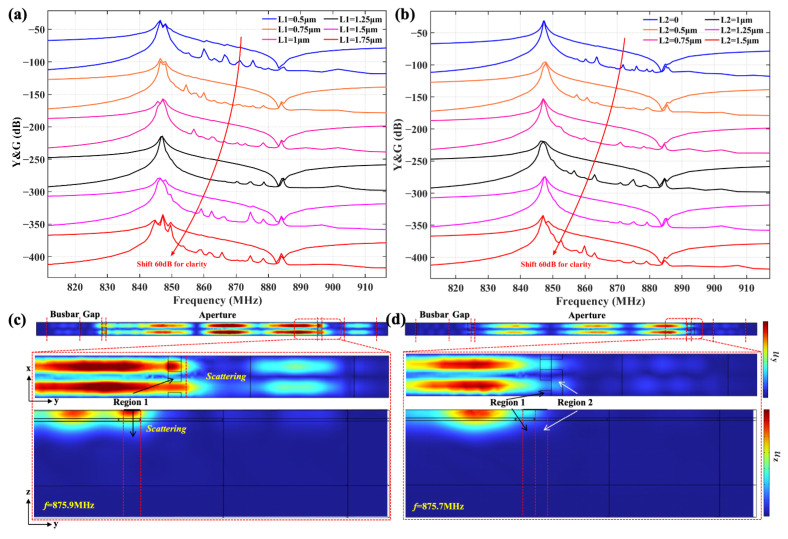
Calculated admittance and conductance curves of TC-SAW resonator with bent metal strip: (**a**) different lengths of *L*_1_; (**b**) different lengths of *L*_2_; the calculated displacement distribution along the *y* and *z* directions. (**c**) *L*_2_ = 0 at 875.9 MHz; (**d**) *L*_2_ = 1.25 μm at 875.7 MHz.

Figure 5a shows the impact of the variation in *L*_1_ on the transverse modes when *h* = 65 nm and *L*_2_ = 1.25 μm. When the length is smaller than 1.25 μm, transverse modes remain visible in the passband, which can be explained according to Equation (1) in that the shorter length of the low-velocity region does not effectively excite the piston mode. As *L*_1_ increases to 1.25 μm, most transverse modes are effectively suppressed. However, with a further increase in *L*_1_ beyond 1.25 μm, transverse modes and a spurious mode below resonance reappear and affect the performance of the device, which indicates that *L*_1_ = 1.25 μm is the optimal length for achieving the best suppression of transverse modes.

Figure 5b shows the variation in the calculated admittance and conductance curves with different lengths of *L*_2_ when *h* and *L*_1_ equal 65 nm and 1.25 μm. The results show that, when the length of *L*_2_ is 0, i.e., the metal strips in region 2 do not exist, the transverse modes and spurious modes near the anti-resonance frequency are obvious. The mitigation of spurious modes gradually improves with an increase in the length of *L*_2_ and reaches the optimum at *L*_2_ = 1.25 μm, and the above spurious modes are effectively mitigated. Then, the mechanism of the mitigation of spurious modes near the anti-resonance is analyzed by field analysis. Figure 5c,d show the calculated displacement distribution along the *y* and *z* directions of spurious modes corresponding to the frequency of 875.9 MHz at *L*_2_ = 0 and 875.7 MHz at *L*_2_ = 1.25 μm in Figure 5b, respectively. Figure 5c shows that, when *L*_2_ = 0, the acoustic wave is reflected by the large velocity barrier, which results in a standing wave field in region 1 and energy scattering into the external gap region [35]. In contrast, the displacement distribution with the addition of the top metal strip in region 2 in Figure 5d shows the reduced energy scattering outside region 1, which can be attributed to a reduction in the velocity barrier between region 1 and the outside region. The reduction in scattering directly corresponds to the calculated curves in Figure 5b, which shows a significant mitigation of the intensity of the spurious mode near the anti-resonance. Based on the above analysis, the length of the external metal strip was determined as *L*_2_ = 1.25 μm in this design.

At the same time, the parameter called the SMSR (Spurious Mode Suppression Ratio) is introduced to quantitatively evaluate the suppression of spurious modes, and a specific explanation of the SMSR is given in the supplemental material. The function relationships between the SMSR value and thickness *h*, length *L*_1_, and length *L*_2_ shown in Figure S2 demonstrate that the optimal parameters (i.e., h=65 nm, $L_{1}=1.25\;\mu m$, and $L_{2}=1.25\;\mu m$) determined by the above calculations are not arbitrary, but in fact correspond to the optimal peaks observed in the SMSR curves. It should be noted that, although the value of the SMSR at *L*_2_ = 1.25 μm is not optimal, the number of spurious modes in the conductance curves of Figure 5b at this value is significantly less than the optimal value of *L*_2_ = 0.75 μm, and therefore, 1.25 μm was chosen as the optimum value of *L*_2_. Therefore, the SMSR analysis quantitatively justifies the chosen parameters as a robust local optimum that effectively suppresses spurious modes.

## 3. Experimental Validation

### 3.1. The Fabrication of TC-SAW Resonators

To verify the validity of the proposed structure in the suppression of spurious modes, a series of SiO_2_/Cu/128°YX-LiNbO_3_ one-port TC-SAW resonators with three structures were fabricated, including the conventional structure, the conventional structure with piston operation, and the proposed structure with bent metal strips on the top of the SiO_2_ layer, where the conventional structure and the conventional piston structure are used for comparison to verify the effectiveness of the proposed schemes.

**Figure 6:**
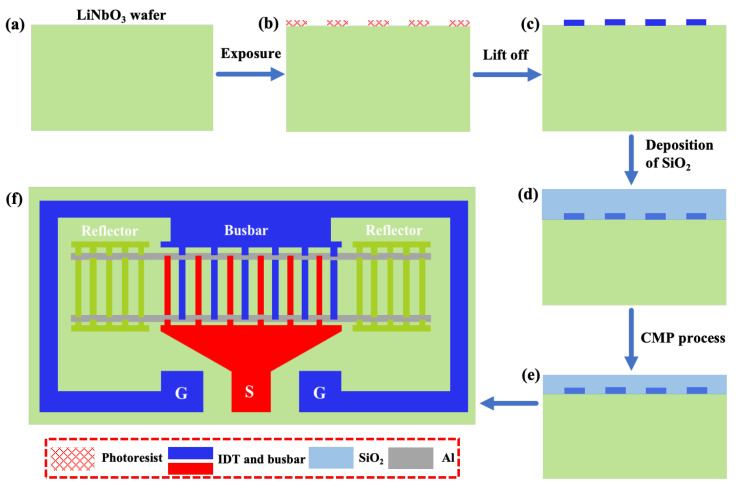
The fabrication process of the TC-SAW resonators. (**a**) Preparation of LiNbO_3_; (**b**) Photolithography process; (**c**) Deposition of IDT electrodes; (**d**) Deposition of SiO_2_; (**e**) Chemical mechanical polishing (CMP) process; (**f**) The schematic diagram of TC-SAW resonator with bent electrodes.

The fabrication process of the TC-SAW resonators with the proposed structure is shown in Figure 6: First, the metal electrode layer consisting of Cr, Ag, Cu, and Cr with thicknesses of 3 nm, 2.5 nm, 307 nm, and 10 nm was formed by a lift-off process on the the surface of a 128°Y-X LiNbO_3_ substrate. The bottom layer of Cr in the multilayered electrode was used as an adhesion layer to enhance the bonding between the IDT electrode and the substrate. The middle layer of Cu was used as the dominant electrode layer, and the introduction of metal Ag could effectively reduce the electrode resistance and ohmic loss, thus enhancing the *Q* factor of the resonator. The top layer of Cr acted as a protective layer, which not only isolated the internal Ag/Cu layer from the external environment, but also effectively prevented it from oxidizing during the subsequent SiO_2_ deposition process. Then, a 330 nm thick SiO_2_ layer was deposited on the 128°Y-X LiNbO_3_ substrate by plasma-enhanced chemical vapor deposition (PECVD). Finally, a bent metal strip layer of aluminum with different geometric parameters was deposited on the SiO_2_ layer by magnetron sputtering, and Figure 6f shows a schematic diagram of the one-port SAW resonator with the proposed structure.

**Figure 7:**
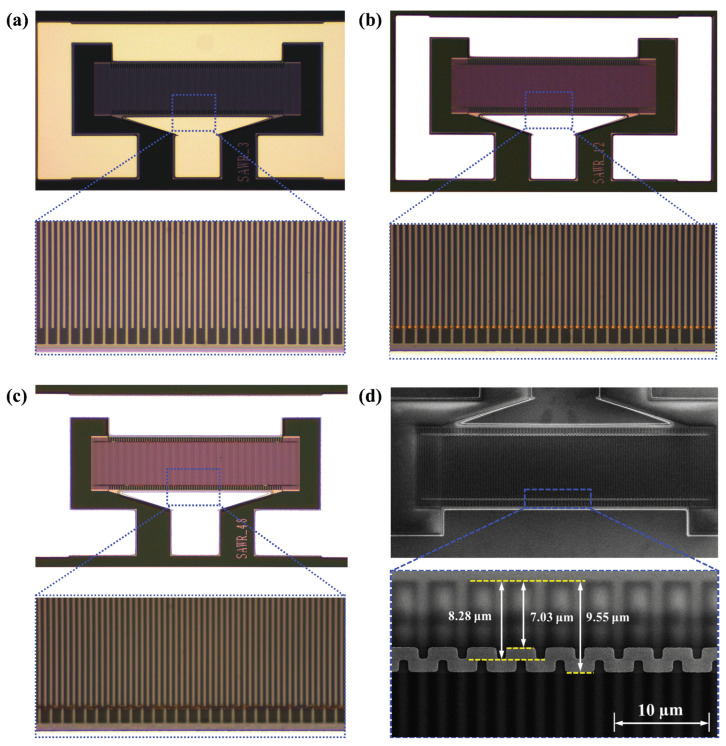
The optical microscope image of the fabricated TC-SAW resonator. (**a**) Conventional structure. (**b**) Conventional structure with piston operation. (**c**) Proposed structure with bent metal strips. (**d**) SEM images of fabricated TC-SAW resonators with bent metal strips.

The optical microscope and SEM microscope images of the fabricated TC-SAW resonator according to the above fabrication process are shown in Figure 7. The optical microscope image in Figure 7a–c shows the overall structure of the fabricated TC-SAW resonator with three structures and the partially enlarged morphology of the edge of the IDT electrodes. The IDT electrodes are arranged regularly, with straight edges and excellent shapes, and there are no obvious defects or broken fingers. The SEM characterization results of the proposed TC-SAW resonator with bent metal strips on the top of the SiO_2_ layer in Figure 7d show that the metal strips are neatly deposited on top of the SiO_2_ with clear outlines. Measurements show that the length of the metal strips in the IDT aperture region, *L*_1_, is approximately 1.27 μm, while the width of the gap region, *L*_2_, is approximately 1.25 μm. All the structural parameters generally meet the optimal design parameters.

### 3.2. Analysis of Fabricated TC-SAW Resonators

**Figure 8:**
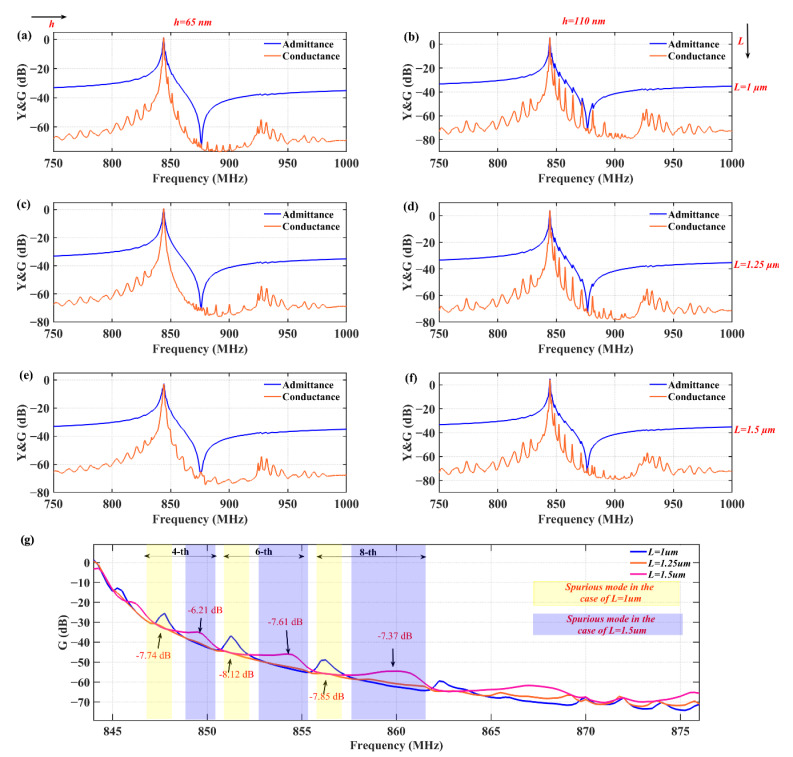
(**a**–**f**) Measured admittance and conductance curves of the TC-SAW resonator with bent metal strip on the top of the SiO_2_ layer under different thicknesses and lengths. (**g**) Variation in several spurious peak amplitudes of conductance when the thickness of the bent metal strip is 65 nm.

The measurement was executed with a ground-signal-ground (GSG) probe connected to a vector network analyzer. Before the formal test, the Short-Load-Open-Through (SLOT) calibration method was pre-adopted in order to exclude parasitic signal interference from equipment, RF cables, and adapters to ensure the accuracy and reliability of the test environment. Figure 8 shows the measured admittance versus conductance curves of the TC-SAW resonator with bent metal strips, which compares the response of the fabricated devices for different geometric parameters of metal strips. The left vertical plots (a), (c), and (e) correspond to the response curves at a top metal strip thickness of 65 nm, and the right vertical plots (b), (d), and (f) show the response curves at a top metal strip thickness of 110 nm. Meanwhile, the response curves of the top plots (a) and (b) and the middle plots (c) and (d), as well as the bottom plots (e) and (f), correspond to lengths of *L*_1_ and *L*_2_ equal to 1 μm, 1.25 μm, and 1.5 μm, respectively. The measured results indicate that the transverse mode can be well suppressed at a thickness of 65nm. Figure 8g shows the effect of the metal strip’s length on the amplitudes of several spurious mode peaks (fourth, sixth, and eighth) when the thickness is 65 nm, and the comparison results show that the amplitudes at ***L*** = 1.25 μm are significantly attenuated compared to in the other two cases. These results confirm that the transverse modes can be effectively suppressed in the TC-SAW resonator using these specific structural parameters (*h* = 65 nm and ***L*** = 1.25 μm), which is consistent with the simulation results.

**Figure 9:**
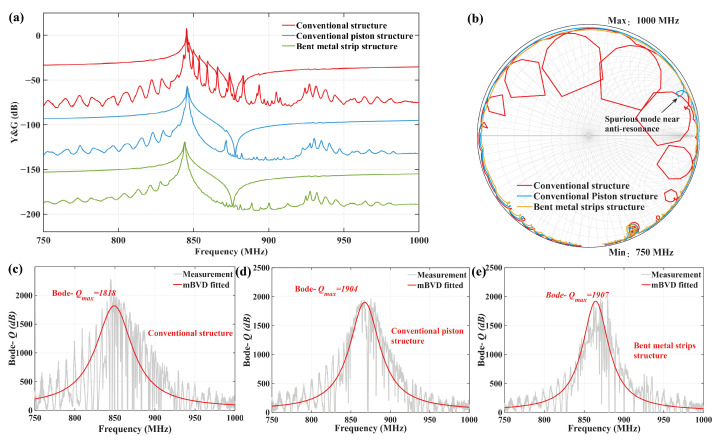
(**a**) The measured admittance and conductance responses of TC-SAW resonators of three different structures; (**b**) Smith chart; (**c**) quality factor of conventional structures; (**d**) quality factor of conventional piston structures; (**e**) quality factor of bent metal strip structure on top of SiO_2_ layer.

Then, the performance of the fabricated TC-SAW resonators with three different structures was compared, including the conventional structure, the conventional structure with piston operation, and the proposed bent metal strip structure with optimal design parameters. Figure 9a shows the measured admittance and conductance response of TC-SAW resonators of three different structures. It can be seen that the transverse modes between *f_s_* and *f_p_* are obvious in the conventional structure. The transverse modes are mostly suppressed in the conventional piston structure, but there is still a spurious mode named the gap mode near the anti-resonance. However, for the proposed structure with optimal bent metal strips on the top of the SiO_2_ layer, nearly all spurious modes are effectively suppressed at the cost of introducing some spurious modes with small amplitude near the anti-resonance. Figure 9b shows a Smith chart of the three different structures, where the blue, orange, and green curves correspond to the conventional structure, conventional piston structure, and proposed structure with bent metal strips on the top of the SiO_2_ layer, respectively. It can be seen that the impedance curve of the conventional structure is very irregular due to the presence of transverse modes. The impedance curve of the conventional piston structure is relatively smooth, but there are still some fluctuations caused by the gap mode near the anti-resonance. However, the impedance curve for the proposed structure is very smooth and regular, without any fluctuations. Then, the quality factors of the three fabricated resonators are analyzed by the Bode-*Q* method. Figure 9c–e show the measured and modified Butterworth-van Dyke (mBVD) equivalent circuit [36,37] fitted Bode-*Q* of fabricated resonators of three different structures, respectively. The Bode-*Q* was calculated by

(3) $$Bode-Q=\frac{2\pi f\left|S_{11}\right|\tau \left(S_{11}\right)}{1-{\left|S_{11}\right|}^{2}}$$

where *f* is the signal frequency and τ represents the group delay. It can be found that the maximum value of the mBVD fitted Bode-*Q* for the proposed structure is 1907, which is basically comparable to the maximum mBVD fitted value for the other two structures without substantial differences. It is also worth noting that the Bode-*Q* value of the conventional piston structure shows a sharp decrease near the anti-resonance frequency (about 870 MHz) due to the gap mode. In contrast, this phenomenon is not observed in the proposed structure. Meanwhile, a performance comparison of several TC-SAW resonators is shown in Table 2, which proves that the proposed structure exhibits a higher *Q* value compared with other techniques such as bent resonator.

## 4. Conclusions

In this work, the suppression of spurious modes in TC-SAW resonators with bent metal strips on the top of a SiO_2_ layer was investigated. Theoretical simulations using 3D FEM revealed that the geometric parameters of the bent metal strips significantly influence the suppression of spurious modes. Optimal parameters were identified, with a metal strip thickness of 65 nm and lengths of 1.25 μm at both the edge of the IDT aperture region and gap regions yielding the best results. Experimental validation confirmed that TC-SAW resonators with the proposed bent metal strip structure exhibited effective suppression of nearly all spurious modes without compromising the quality factor. This approach offers a promising solution for improving the performance of TC-SAW resonators in RF applications.

## Figures and Tables

**Table 1:** Simulation parameters.

Parameter	Value
Piezoelectric material	128°YX-LiNbO_3_
Electrode material	Cu
Period (λ)	4.14 μm
Metal ratio (MR)	0.5
Thickness of electrode (*h*_Cu_)	0.08λ
Thickness of silicon oxide (*h*_SiO_2__)	0.34λ
Aperture (*W*)	20λ
Gap length (*L*_gap_)	2λ
Busbar length	3λ
PML layer	λ

**Table 2:** Comparison of TC-SAW resonators’ performance.

Type	Frequency	Structure	$Q_{\max}$	Reference
SiO_2_/128°YX-LiNbO_3_	875 MHz	Piston mode apodization	1700 1200	[17]
SiO_2_/128°YX-LiNbO_3_	1935 MHz	Bent resonator	About 1500	[13]
SiO_2_/128°YX-LiNbO_3_	About 1.7GHz	Double metal dots	About 1300	[25]
SiO_2_/128°YX-LiNbO_3_	845 MHz	Bent metal strip	1900	This work

## Data Availability

The data presented in this study are available on request from the corresponding author.

## Supplementary Materials

The following supporting information can be downloaded at: https://www.mdpi.com/article/10.3390/s25226926/s1. Figure S1: The schematics of the $G_{\mathrm{main}}$ and $G_{\mathrm{spurious}\_\max}$ in the conductance curve; Figure S2: The SMSR value as a function of thickness *h*, length $L_{1}$ and length $L_{2}$.
