# Supplementary material for: The Suppression of Spurious Modes in TC-SAW Resonators by the Application of Bent Metal Strips
Source: Sensors (Basel). 2025 Nov 13;25(22):6926. doi: 10.3390/s25226926 (PMC12655950; doi:10.3390/s25226926)
Supplement: Supplementary file 1 [file sensors-25-06926-s001.zip › sensors-3925987-supplementary.pdf]

Supplemental material

The Suppression of Spurious Modes in TC-SAW Resonators by the Application of Bent Metal Strips

Menghui Li[1], Mengke Qi[1], Yuanhang Chen[1], Yimin Cheng[1], Liang Cao[1], Hong Zhou[2], and Xiaojing Mu[1,*]

[1] *Key Laboratory of Optoelectronic Technology and Systems of Ministry of Education, International Research and Development Center of Micro-Nano Systems and New Materials Technology, Chongqing University, Chongqing 400044, China*

[2] *Ministry of Education Key Laboratory of Micro and Nano Systems for Aerospace, School of Mechanical Engineering, Northwestern Polytechnical University, Xi'an 710072, China*

1 The definition of SMSR

The SMSR is defined as $SMSR = G_{\text{main}}(dB)/G_{\text{spurious\_max}}(dB)$, where $G_{\text{main}}(dB)$ is the relative hight of the conductance value of main resonance and $G_{\text{spurious\_max}}(dB)$ is the relative height of the conductance value of strongest spurious (transverse) mode. The SMSR values are extracted from the conductance curve for every parameter sweep (h, L_1, and L_2) and the schematics of the $G_{\text{main}}(dB)$ and $G_{\text{spurious\_max}}(dB)$ in the conductance curve is shown in Figure S1 below. It should be noted that the conductance values are expressed in the formula in $20\log_{10}(G)$, so the SMSR can be calculated as:

$$\begin{aligned}\text{SMSR (dB)} &= G_{\text{main}}(dB)/G_{\text{spurious\_max}}(dB) \\ &= 20\log_{10}(G_{\text{main}})/20\log_{10}(G_{\text{spurious-max}}) \\ &= G_{\text{main}}\ \text{(dB)} - G_{\text{spurious\_max}}\ \text{(dB)}\end{aligned} \tag{1}$$

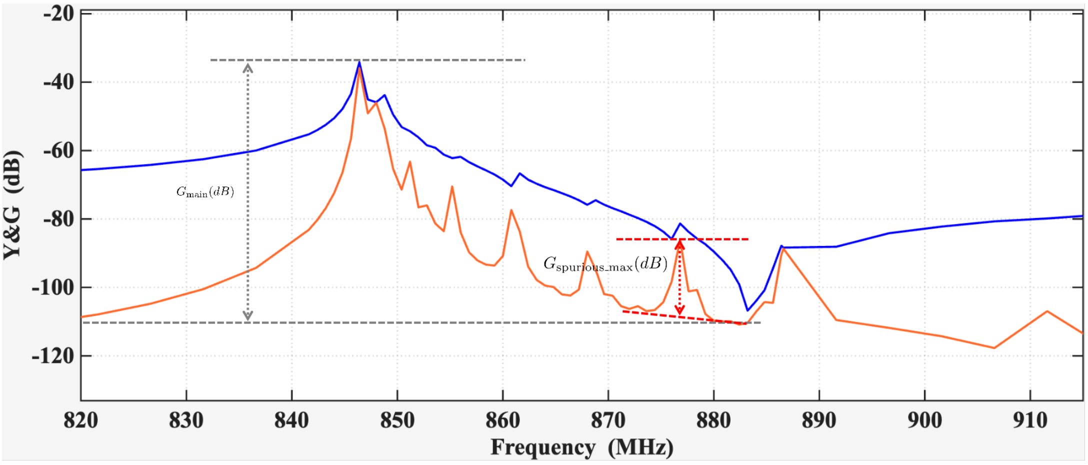

Figure S1. The schematics of the G_{main} and $G_{\text{spurious\_max}}$ in the conductance curve.

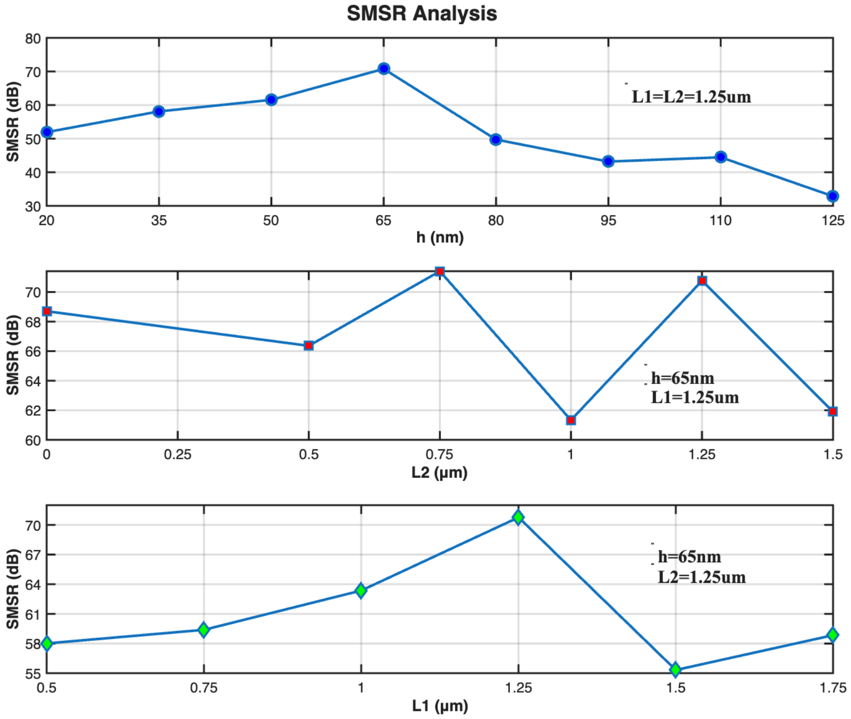

Figure S2. The SMSR value as a function of thickness h, length L_1 and length L_2.

Figure S2 shows the SMSR values extracted from the conductance curves as a function of each parameter (h, L_1, and L_2). These plots quantitatively demonstrate that the optimal parameters (i.e., $h \approx 65$ nm, $L_1 \approx 1.25\mu$m, and $L_2 \approx 1.25\mu$m) are not arbitrary, but in fact correspond to the optimal peaks observed in the SMSR curves. It should be noted that although the value of SMSR at $\boldsymbol{L_2}$= 1.25μm is not optimal, the number of spurious modes in the conductance curves of Figure 5(b) at this value is significantly less than the optimal value of $\boldsymbol{L_2}$= 0.75μm, and therefore 1.25μm was chosen as optimum value of $\boldsymbol{L_2}$. While we acknowledge this 1D decoupled optimization cannot entirely rule out other local optima (which would require the contour plot analysis), the SMSR analysis quantitatively justifies our chosen parameters as a robust local optimum that effectively suppresses spurious modes.
